# Nasal Administration and Plasma Pharmacokinetics of Parathyroid Hormone Peptide PTH 1-34 for the Treatment of Osteoporosis

**DOI:** 10.3390/pharmaceutics11060265

**Published:** 2019-06-07

**Authors:** Richard G. Pearson, Tahir Masud, Elaine Blackshaw, Andrew Naylor, Michael Hinchcliffe, Kirk Jeffery, Faron Jordan, Anjumn Shabir-Ahmed, Gareth King, Andrew L. Lewis, Lisbeth Illum, Alan C. Perkins

**Affiliations:** 1 Division of Rheumatology, Orthopaedics and Dermatology, School of Medicine, Queen’s Medical Centre, University of Nottingham, Nottingham NG7 2UH, UK; 2 Nottingham University Hospitals NHS Trust, Queen’s Med Centre, University of Nottingham, Nottingham NG7 2UH, UK; tahir.masud@nuh.nhs.uk; 3 Radiological Sciences, School of Medicine, Queen’s Medical Centre, University of Nottingham, Nottingham NG7 2UH, UK; elaine.blackshaw@nottingham.ac.uk (E.B.); alan.perkins@nottingham.ac.uk (A.C.P.); 4 Critical Pharmaceuticals Ltd., Bio City, Pennyfoot Street, Nottingham NG1 1GF, UK; anaylor@upperton.com (A.N.); kjeffery@upperton.com (K.J.); faron.jordan@hotmail.co.uk (F.J.); anjumnshabir@yahoo.com (A.S.-A.); gareth.king@catapult-ventures.com (G.K.); andrew.lewis@quotientsciences.com (A.L.L.); 5 Paracelsis Ltd., BioCity Nottingham, Pennyfoot Street, Nottingham NG1 1GF, UK; mh@paracelsis.com; 6 Identity, 19 Cavendish Crescent North, The Park, Nottingham NG71BA, UK; lisbeth.illum@illumdavis.com

**Keywords:** PTH 1-34, teriparatide, nasal delivery, pharmacokinetics, osteoporosis, man, sheep, clinical trial, preclinical

## Abstract

Nasal delivery of large peptides such as parathyroid 1-34 (PTH 1-34) can benefit from a permeation enhancer to promote absorption across the nasal mucosa into the bloodstream. Previously, we have published an encouraging bioavailability (78%), relative to subcutaneous injection in a small animal preclinical model, for a liquid nasal spray formulation containing the permeation enhancer polyethylene glycol (15)-hydroxystearate (Solutol^®^ HS15). We report here the plasma pharmacokinetics of PTH 1-34 in healthy human volunteers receiving the liquid nasal spray formulation containing Solutol^®^ HS15. For comparison, data for a commercially manufactured teriparatide formulation delivered via subcutaneous injection pen are also presented. Tc-99m-DTPA gamma scintigraphy monitored the deposition of the nasal spray in the nasal cavity and clearance via the inferior meatus and nasopharynx. The 50% clearance time was 17.8 min (minimum 10.9, maximum 74.3 min). For PTH 1-34, mean plasma C_max_ of 5 pg/mL and 253 pg/mL were obtained for the nasal spray and subcutaneous injection respectively; relative bioavailability of the nasal spray was ≤1%. Subsequently, we investigated the pharmacokinetics of the liquid nasal spray formulation as well as a dry powder nasal formulation also containing Solutol^®^ HS15 in a crossover study in an established ovine model. In this preclinical model, the relative bioavailability of liquid and powder nasal formulations was 1.4% and 1.0% respectively. The absolute bioavailability of subcutaneously administered PTH 1-34 (mean 77%, range 55–108%) in sheep was in agreement with published human data for teriparatide (up to 95%). These findings have important implications in the search for alternative routes of administration of peptides for the treatment of osteoporosis, and in terms of improving translation from animal models to humans.

## 1. Introduction

Fragility fractures are associated with osteoporosis, a skeletal disease that occurs mainly in the older population, and are associated with a reduced bone mineral density (BMD), with alterations in bone microarchitecture such that the bone exhibits a reduced capacity to resist fracture [1,2]. Osteoporosis is a significant contributor to non-traumatic hip fractures and vertebral fractures worldwide. In 2017, there were 66,668 hip fractures in those aged 60 years or over in the UK alone. The human cost in this patient group is high, with a mortality rate of 6.7%. A 30 day survival for patients with hip fracture can be accurately estimated using the Nottingham Hip Fracture Score [3,4]. This is mirrored by a huge health economic burden, and costs for the NHS and social care of £1 billion per year in the UK [5].

PTH 1-34 injections (teriparatide) are currently the only anabolic therapy specifically designed for the treatment of osteoporosis by promoting the deposition of bone to increase bone density as opposed to preventing bone loss. Poor compliance of between 25–30% at 12 months is widely reported in patient populations prescribed teriparatide treatment [6,7]. When this patient group is questioned, there is a reluctance to be prescribed teriparatide due to the need for repeated injection [8]. Therefore, we are researching nasal spray formulations as an alternative to daily injection, as a strategy for improving patient compliance which could afford improved patient outcomes and reduced economic healthcare/social care burden. In addition, there could be economic benefits due to reduced manufacturing costs.

Nasal spray delivery systems can facilitate easy, painless administration of a drug, and are convenient for patients to self-administer. However, large hydrophilic molecules such as peptides and proteins are poorly transported across the nasal membrane, and hence require the use of a permeation enhancer to achieve a therapeutically relevant bioavailability. Furthermore, it can be a challenge to develop a suitable nasal formulation since many permeation enhancers are poorly tolerated by the nasal membrane [9,10,11]. Enabling technologies in the form of both nasal delivery devices and nasal mucosal permeation enhancers are presently being developed for effective nasal delivery of peptides/proteins. Such technologies are being pursued in clinical trials [12,13]. The nasal delivery system investigated here contains a permeation enhancer aimed at promoting absorption of the PTH 1-34 (4.1 kDa 34 N-terminal amino acid peptide) across the nasal mucosa in a safe manner [9]. The selected permeation enhancer is a pharmaceutical excipient, polyethylene glycol (15)-hydroxystearate, also known as Macrogol (15)-hydroxystearate, polyoxyethylated 12-hydroxystearic acid, Solutol^®^ HS 15, Kolliphor^®^ HS 15 or CriticalSorb™ [10].

The therapeutic efficacy of the intranasal spray formulation of PTH 1-34 is dependent upon pharmacokinetics. To investigate this, the rat preclinical model was previously utilised, and showed that serum levels of PTH 1-34 were significantly greater when delivered in nasal formulations containing Solutol^®^ HS15, compared to a simple PTH 1-34 formulation [14]. We have also previously demonstrated that Solutol^®^ HS15 is an essential excipient for the enhancement of human growth hormone delivery via the intranasal route [10]. Solutol^®^ HS15, a mixture of mono- and diesters of 12-hydroxystearate (macrogol 15-hydroxystearate), is a non-ionic surfactant with a hydrophilic–lipophilic balance value of 14–16. Due to its amphiphilic nature, Solutol^®^ HS15 in solution forms micelles approximately 13 nm in diameter. Importantly, when peptides or proteins are dissolved in an aqueous solution comprising Solutol^®^ HS15, they retain their tertiary structure [15]. The mechanism of action of Solutol^®^ HS15 is considered to arise primarily from a combination of effects on the cell membrane (transcellular enhancement mechanism), and from an impact on the organization of the actin in the cell cytoskeleton resulting in tight junction opening (paracellular enhancement mechanism). Solutol^®^ HS15 enhances the transport of drugs across the mucosal membrane without demonstrating toxicity to mucosal tissue [9]. The formulation is compliant with the European Medicines Agency guidelines on pharmaceutical quality of inhalation and nasal products (EMEA/CHMP/QWP/49313/2005).

Our previously published preclinical data in rats for a liquid nasal spray formation were encouraging, showing that the nasal systemic absorption of PTH 1-34 was increased considerably when administered in a formulation containing Solutol^®^ HS15. The mean C_max_ obtained for the nasal spray (13.7 ng/mL after administering 100 μg/kg PTH 1-34) was comparable to that after subcutaneous injection (14.8 ng/mL after a 80 μg/kg dose), and the relative bioavailability was 78% [14]. These data led to the initiation of the clinical study assessing the liquid nasal spray formulation. This was carried out to assess nasal deposition, clearance and pharmacokinetics in human subjects [16]. In addition, on review of the clinical data obtained, we followed up this research using a large animal (ovine) model reported to be reliable and predictive of human intranasal drug delivery [17]. This replicated the evaluation of the liquid nasal spray formulation that was given to healthy human volunteers, in addition to piloting an intranasal dry powder formulation containing the same absorption enhancer excipient [18].

## 2. Materials

The PTH 1-34 acetate used for the nasal formulation was chemically synthesised (Polypeptide Inc., Torrance, CA, USA), and recombinant PTH 1-34 known as teriparatide was the active formulation ingredient (API) in the commercially available subcutaneous injection pen, Forsteo^®^, obtained from Lilly France S.A.S, Fegersheim, France. HyPure WFI Quality Water (Ph.Eur.) (Thermo Fisher Scientific, Loughborough, UK), Solutol^®^ HS15 (BASF, Ludwigshafen, Germany). All other materials were supplied by Sigma Aldrich, Gillingham, UK: glacial acetic acid USP, sodium acetate USP, d-mannitol (Ph.Eur.) and l-methionine (Ph.Eur.). Gellan gum, Gelzan™ CM (CP Kelco), and trehalose USP Tc-99m-DTPA (Diethylenetriamine pentaacetate) supplied by the radiopharmacy unit of Queen’s Medical Centre, University of Nottingham.

## 3. Methods

### 3.1. Preparation of Nasal Formulations for Clinical Study

The nasal formulation comprised 0.1% *w*/*v* PTH 1-34 (Polypeptide Inc., Torrance, CA, USA), 7.5% *w*/*v* polyethylene glycol (15)-hydroxystearate (Ph.Eur.), 5% *w*/*v* d-mannitol (Ph.Eur.), 3% *w*/*v* l-methionine (Ph.Eur.), 0.15% sodium acetate (Ph.Eur.), and 0.8% *w*/*v* acetic acid (Ph.Eur.) in water for injection (Ph.Eur.) containing the radiolabel (Tc-99m-DTPA). To achieve a 90 µg dose of PTH 1-34, 90 µL of the formulation was atomised in each actuation using a Standard Rexam 3959 winged spray actuator, SP270-90 pump (Rexam Healthcare, London, UK). In brief, for manufacture of the radiolabel (Tc-99m-DTPA) containing formulation, Solutol^®^ HS15 was rendered molten by raising the temperature to 60 ± 10 °C. Molten Solutol^®^ HS15 was then dissolved in 0.2 M acetate buffer (pH 4.0) containing the d-mannitol and l-Methionine at 50 ± 5 °C. The resulting 11% *w*/*v* Solutol HS15 was allowed to cool and was used to prepare the 1 mg/mL PTH 1–34, Tc-99m-DTPA (3–5 MBq activity), 7.5% Solutol^®^ HS15 formulation in 0.1 M acetate buffer pH4 using water for injection (Ph.Eur.) as the diluent. Preparation of the formulations was performed at the GMP facility at the Queen’s Medical Centre, Nottingham, UK.

The droplet size from the nasal spray was assayed for a 45 µL dose volume by Rexam (Suresnes, France) using a Spraytec (Malvern Panalytical, Malvern, UK) instrument, activation speed: 60 mm/s, acceleration: 2500 mm/s^2^, with a symmetric profile. All parameters were analysed during the stabilization phase of the spray, with actuator positioned 6 cm from the laser. D10: Droplet diameter such that 10% of the total liquid volume consist of droplets of smaller diameter (in μm), similarly for D50 and D90. SPAN is a measure of the droplet size distribution and is defined by SPAN = (D90 − D10)/D50. D10 = 29.2 (26.8–33.5) μm, D50 = 59.7 (51.4–75.3) μm, D90 = 127.3 (102.7–155.6) μm, SPAN 1.64 (1.48–1.76).

A HPLC based analytical method was used to determine stability of the nasal formulation and was defined as 97.0–103.0 percent (*n* = 4) of the initial drug concentration in the formulation following storage (2–8 °C) over a period of 48 h, the maximum time elapsed between manufacture and dosing of the patients. Column: Discovery Bio wide pore C18 (25 mm × 4.6 mm i.d., 5 μm); Mobile Phase: Phase A: 0.1 M Sodium Perchlorate, pH 2.7, Phase B: Acetonitrile; Flow Rate: 1.0 mL/min; Run Time 50 min; Injection Volume: 15 μL; Column Temperature: 60 °C; Auto Sampler Temperature: 2–8 °C; Detection: 210 nm.

### 3.2. Preparation of Nasal Formulations for Ovine Study

The liquid nasal formulation used in the sheep was prepared identically to that for the human study, with the exception that it was manufactured in the Critical Pharmaceutical Laboratories (CPL) and did not contain the Tc-99m-DTPA label. The dry powder formulation was also prepared at CPL as 1% *w*/*w* PTH (1-34) with 39% Gellan gum, 40% Solutol^®^ HS15, 20% Trehalose. In brief, 97.5 mg Gellan gum was dissolved in 50 mL ultra-pure water overnight. Solutol^®^ HS15 was rendered molten (60 ± 10 °C), and 100 mg was added to the Gellan gum solution and mixed for 30–45 min at 60 °C. 50 mg of trehalose was added and mixed for a further 10 min. A total 5 mg of PTH 1-34 was dissolved in 5 mL of ultra-pure water, and 2.5 mL was added to the formulation. Particles were prepared using a spray dryer Mini Spray Dryer B-290 (Buchi UK Ltd., Oldham, UK) with the inlet set at 85 °C (outlet modified by inlet temperature), the aspirator 100%, the pump set at 006 equivalent to 2 mL/ min, the spray pressure at 4 bar. Particle size of the dry powder formulation manufactured for use in the sheep was not measured, due to limited availability of material due to batch size. However, the particle size of similar formulations when prepared for loading into the delivery devices was monitored using Helos/BF (Sympatec GmbH, Clausthal-Zellerfeld, Germany) during formulation development, where the VMD was 73.7–91.8 µm (×10 = 10.6–23.7 µm, ×50 = 51.5–98.1 µm, ×90 = 142–153 µm). A total 2.4–2.8% of particles were below 10 µm. As the vast majority of particles were >10 µm, the formulations would be expected to be nasally deposited. The integrity of the spray dried PTH 1-34 was monitored during formulation development by HPLC analysis, which consistently revealed a purity of >82.5% PTH 1-34.

### 3.3. Healthy Human Volunteer Pharmacokinetic/Gamma Scintigraphy Study

The study was conducted in accordance with the Declaration of Helsinki, and the protocol for the clinical study was given a favourable opinion (12 September 2013) by the NRES committee London Westminster (ref 13/LO/1037, IRAS ID 126447). All participants gave their informed consent before they participated in the study. This included regulatory approvals from the Medicines and Healthcare products Regulatory Agency (MHRA), Nottingham University Hospitals NHS Trust Research & Innovation (NUH NHS Trust R&I) and certification from the Administration of Radioactive Substances Advisory Committee (ARSAC). The study was registered at https://clinicaltrials.gov/ website and with the Trent Comprehensive Local Research Network (Trent CLRN). A dose escalation component was included within the approved study using a developmental delivery device, however, the data are not reported here due to commercial sensitivities.

The focus of the study was to investigate the plasma pharmacokinetics of parathyroid hormone PTH 1-34 delivered in a liquid nasal spray formulation containing the excipient polyethylene glycol (15)-hydroxystearate (Solutol^®^ HS15). Furthermore, the nasal deposition and clearance of the formulation was investigated using gamma scintigraphy. The pharmacokinetics and the deposition and clearance of the nasal spray by gamma scintigraphy were investigated in the same clinical study.

#### 3.3.1. Pharmacokinetics

An open cross-over clinical IMP (investigative medicinal product) study was conducted in seven healthy female subjects aged over 55 years (mean age 67.7, range 58 to 81 years). Each participant provided informed consent in accordance with ethical committee requirements and good clinical practice. Pre-study screening assessments were carried out within 21 days of the participants receiving their first dose (Supplementary Materials: Section S1). Inclusion and exclusion criteria were applied (Supplementary Materials: Section S2). Participants were admitted to the study centre on the morning of dosing, having abstained from alcohol and smoking for at least 24 h previously and fasted from midnight. Participants were given a light meal on arrival. A cannula was then positioned in the participant’s arm or hand to facilitate the collection of blood samples. The pre-dose sample was taken. Participants received a single subcutaneous injection of 20 μg teriparatide into the abdomen at their first trial visit. Following a seven day wash out period, each participant self-administered 90 μg PTH 1-34 from the nasal spray formulation using the Rexam SP270 nasal spray device; the patients had previously been trained in operation of the Rexam device. Any episodes of sneezing after administration of the nasal formulation were recorded. The nasal spray formulation contained Tc-99m-DTPA, 3–5 MBq per Rexam SP270 nasal spray dose, resulting in an effective radiation dose of less than 0.14 mSv to each participant.

Following either subcutaneous or intranasal dosing, 10 mL blood samples (Becton Dickinson sodium heparin) were taken at 5, 15, 30, 60 120, 180, 240, 300 and 360 min. After each sample was taken, the cannula was flushed with 10 mL 0.9% saline. Blood was stored on ice and centrifuged within 30 min to prepare plasma (4 °C, 1100–1300 g, 10 min). These samples were stored at −20 °C before dispatching to Simbec Research Ltd. (Merthyr Tydfil, UK) for quantification by validated ELISA, in compliance with The UK Good Laboratory Practice Regulations 1999 (Statutory Instrument No. 310 6) and subsequent amendment, OECD Principles of Good Laboratory Practice (Paris 1998) and EC Commission Directive 2004/10/EC of February 2004.

Pharmacokinetic data were analysed using Phoenix^®^ WinNonlin^®^ 5.1 (Certara USA, Inc., Princeton, NJ, USA) from quantified PTH 1-34 in the plasma, using recorded sampling times. Summary statistics (mean, median, SD, coefficient of variation [CV%], minimum, maximum, *n*) and the following plasma pharmacokinetic parameters were calculated:

- C_max_
- T_max_
- Terminal half-life (t_1/2_)
- AUC to 2 h (AUC_0–2h_)
- AUC to last measured time point (AUC_last_) and corresponding F_rel_ for nasal formulation

#### 3.3.2. Gamma Scintigraphy

For the gamma scintigraphy part of the study, a custom designed, rigid frame was attached to a single head X ring gamma camera (Mediso Ltd., Budapest, Hungary) and used to support the chin of the participant. This maintained a fixed position of the nose in a sagittal view relative to the camera, and negated the need to register the longitudinal image series. From immediately post-dose, a 20 min continuous (dynamic) image acquisition was recorded comprising 40 × 30 s frames. In addition, 30 min post administration, a 30 s frame was acquired which was repeated every 30 min thereafter, terminating at 2 h post-dose. Participants returned for a safety follow-up between 1 and 14 days after their final study procedure.

All seven participants attended the University of Nottingham MRI clinical facility (Sir Peter Mansfield Imaging Centre, School of Medicine) prior to the clinical study to generate a sagittal view of the head and neck to provide an anatomical image of the nasal cavity. The acquisition sequence protocol was: 3 plane localiser, Asset Calibration, Coronal STIR, Sagittal T_2_ and Sagittal T_1_ using a GE 750 3T MRI fitted with a 32 channel head coil.

Using a HERMES image workstation (Hermes Medical Solutions, Gravesend, UK) the scintigraphic images were superimposed upon the DICOM MRI images to enable reference of the nasal spray deposition site and to define formulation clearance relative to anatomical position within the nasal cavity. Using the sagittal MRI anatomical view, four regions of interest (ROI) were defined: one around the anterior site of deposition in the nasal cavity, the second covering the nasal cavity and nasopharynx to the level of the oropharynx, a third covering the entire region and a fourth distal region to obtain the background counts. The counts obtained from the ROIs were subject to various corrections to account for image time, background activity and radioactive decay. The time for 50% clearance of the total amount of radioactivity deposited in the nasal region (T_50%_) was calculated for each subject. T_50%_ was calculated from corresponding activity–time profiles by fitting 4 parameter logistic regression (GraphPad Prism 6). For data where this program was unable to make a satisfactory curve fit, the T_50%_ was obtained by straight line interpolation.

#### 3.3.3. Ovine Study

Research was conducted in accordance with the requirements of the Animals (Scientific Procedures) Act 1986 (ASPA) under Procedural Project Licence (PPL) number 40/3552 (Protocol 19b/1), between 26 April 2016 and 24 May 2016, at the University of Nottingham, Nottingham, UK (Procedural Establishment Licence (PEL) number PCD 40/2406). The overarching protocol (as detailed in the PPL) was subject to full review by the Animal Welfare and Ethical Review Board (AWERB), and the study-specific protocol received a more localised review by the Named Animal Care and Welfare Officer (NACWO) and Named Veterinary Surgeon (NVS). This research governance is compliant with recommended ethical review processes. In view of the clinical data obtained in the present study, the pharmacokinetics of the Solutol^®^ HS15-based formulation of PTH 1-34 were further investigated in sheep. The sheep study replicated the clinical trial in that it tested the nasal solution formulation of PTH 1-34 as well as a subcutaneous injection, but also evaluated pharmacokinetics of PTH 1-34 following intravenous injection and an intranasal PTH1-34 dry powder formulation.

The study was conducted according to a four-way randomised cross-over study in four (Mule crossbred) female sheep (obtained from a reputable commercial supplier). The sheep were group housed in a heat and ventilation air conditioned (HVAC) facility for the duration of the study, and were uniquely identified by a combination of ear tag and electronic transponder. On arrival, the animals were also randomly assigned a unique identifier by the facilities Research Animal Facility Management system (LabTracks, Locus Technology Inc., Manchester, MD, USA) and an abbreviated study number (1, 2, 3 and 4). To aid identification during the study, the fleece of each animal was marked with the abbreviated study number by spraying with a commercial stockmarker (dye). The animals were acclimatised for 17 days prior to initiation of treatments. The animals were subject to a health inspection by the NVS prior to and after use in the study, and were monitored during the study by the PPL holder and facility personnel supported by the NACWO, NVS and University of Nottingham personnel. Animals were weighed weekly during the study (Supplementary Materials: Section S3).

The sheep were housed in a temperature and humidity controlled facility (14.2–22.2 °C and humidity from 42–70%) for the duration of the study. On the four study legs, temperature and humidity during the experimental period ranged from 17.6–19.8 °C and 51–61%, respectively. Sheep were routinely fed twice daily with hay and standard grower diet (pellets), with water ad libitum. The sheep were provided with food (approximately double rations of hay) to last overnight on the day prior to each study leg; remaining food was removed approximately 60 min before the start of dosing on each leg. Water was available throughout. Sheep were fed as normal following collection of the last blood sample on each study leg. At the end of the study, the animals were euthanised by overdose of anaesthetic.

Administration of the PTH 1-34 was either as an intranasal liquid, intranasal dry powder, subcutaneous injection or intravenous injection. The treatments were randomised according to a Latin Square design using an online randomisation programme (Experimental Design Generator and Randomiser (Edgar) accessed from: http://www.edgarweb.org.uk/) with a minimum of a two day washout period between study legs. Immediately prior to dosing, the four sheep were sedated by an intravenous injection of ketamine hydrochloride (100 mg/mL solution administered at 2.25 mg/kg). This provided approximately 3 min sedation, during which period the sheep were dosed.

The formulations were prepared at Critical Pharmaceuticals’ Laboratories and transported, refrigerated, to the study facility. The intranasal liquid was administered via the right nostril using a LMA™ MAD110 Nasal™ Intranasal mucosal atomization device (MAD110) (Wolfe-Tory Medical, Inc., Salt Lake City, UT, USA) customised for use in sheep (to allow circumvention of the nostril and delivery of the dose to the respiratory mucosa of the nasal cavity). The intranasal powder formulation was also administered via the right nostril using a 5 mm oral/nasal tracheal tube Portex^®^ Blueline 100/111/050, (Smiths Medical, Ashford, UK) and one-way bellows. The amount of powder administered to each animal, and thus the nominal dose of PTH 1-34, was determined by weighing the devices before and after the nasal administrations. The subcutaneous and intravenous bolus injections of PTH 1-34 were administered into the flank and a jugular vein, respectively. Further details relating to dose administration are provided in Table 1. It has been reported in the literature that injections into the thigh as opposed to the abdomen have been estimated to result in a 21% slower rate of absorption, resulting in an 18% reduction in maximum plasma teriparatide concentrations (C_max_) and 1.5 min prolongation in the time to reach peak concentration (T_max_) [19]. Therefore, care was taken to mirror the subcutaneous injection site (flank) in the sheep model.

A qualitative assessment of local tolerability to the administered intranasal formulations (animals dosed by injection served as controls) was made by recording the incidences of sneezing/snorting in the first 60 min following dosing and any evidence of nasal discharge prior to dosing, at 15 min intervals in the first 60 min, and then at subsequent blood sampling time points thereafter. As part of the monitoring procedure, any other ‘remarkable’ observation was noted.

For pharmacokinetic evaluation, blood samples (3 mL) were collected by direct venepuncture of a cephalic vein (both veins used interchangeably) under local anaesthesia (topical application of EMLA™ Cream 5%, Astra Zeneca). For intranasal and subcutaneous treatments, blood samples were collected at time 0 (prior to dosing), 5, 10, 15, 30, 45, 60, 120, 180 and 240 min post-dose administrations. For the intravenous treatment, blood samples were collected at time 0 (prior to dosing), and at 2, 5, 15, 30, 45, 60, 120, 180 and 240 min post-dose administration. Each blood sample was dispensed into a BD Vacutainer and stored on crushed ice prior to centrifugation at 2000× *g* (3135 rpm) for 10 min at 4 °C (Sorvall™ legend RV refrigerated centrifuge). The samples were stored at −20 °C prior to PTH 1-34 quantification by ELISA kit (Immunotopic, San Clemente, CA USA) as directed by the manufacturer; optical densities were measured using an LT-4000 Microplate Reader (Labtech International Ltd, Heathfield, UK). 

Pharmacokinetic analysis of the plasma PTH 1-34 concentration–time data was performed using Phoenix^®^ WinNonlin^®^ 6.4 (Certara USA, Inc., Princeton, NJ, USA) and Excel 2016 (Microsoft, Redmond, WA, USA). The following were used as the principal measures to evaluate intranasal and subcutaneous absorption of PTH 1-34 in the sheep compared to intravenous injection: C_max_; T_max_; t_1/2_; AUC_last_; and AUC_INF_ (definitions as before); C_0_ (initial plasma concentration estimated by back-extrapolating from the first two concentration values) was also estimated after intravenous administration only. F_rel_ (bioavailability of intranasal (IN) or intravenous (IV) PTH 1-34, relative to subcutaneous (SC) injection) and F_ab_ (absolute bioavailability of IN or SC PTH 1-34, relative to IV) were estimated as follows:

F_rel_ (%) = (AUC_INF(IN or IV)_ × Dose_(SC)_) ÷ (AUC_INF(SC)_ × Dose_(IN or IV)_) × 100

F_ab_ (%) = (AUC_INF(IN or SC)_ × Dose_(IV)_) ÷ (AUC_INF(IV)_ × Dose_(IN or SC)_) × 100

## 4. Results

### 4.1. Clinical Study

All seven healthy volunteers completed the clinical study and all pre- and post-clinical assessments were acceptable. There were no serious adverse events during this study and no participant sneezed after nasal dosing.

#### 4.1.1. Pharmacokinetics

**Figure 1:**
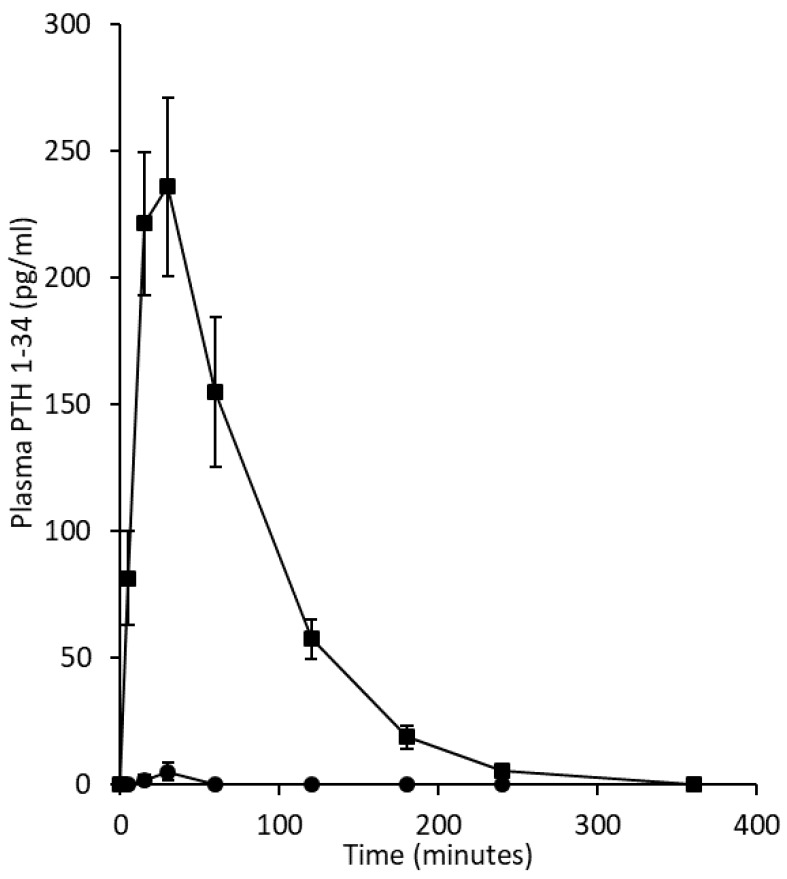
Pharmacokinetics of a single dose of either a liquid intranasal spray formulation containing the excipient Solutol^®^ HS15 or a subcutaneous injection of teriparatide by a pen to healthy female subjects aged over 55 years (mean age 67.7, range 58 to 81 years). Circles represent the mean ± SE of the PTH 1-34 plasma concentration delivered from a Rexam SP270 nasal spray device (*n* = 7). Squares depict the PTH 1-34 plasma concentration in participants following subcutaneous injection using a commercially available formulation and injection pen (Eli Lilly).

The mean pharmacokinetic profiles of PTH 1-34 when delivered as a liquid nasal spray formulation or as a subcutaneous injection to healthy volunteers and corresponding summary pharmacokinetic data are presented in Figure 1 and Table 2, respectively. The mean profile obtained following subcutaneous administration (20 μg dose) of teriparatide was consistent with those obtained in individual subjects (not shown) and showed rapid absorption (mean C_max_ 253 pg/mL with range in C_max_ values of 141.8 to 406.5 pg/mL and mean T_max_ of 21 min) and elimination (mean t_1/2_ approximately 1 h). Teriparatide concentrations returned to zero by 6 h. This was in contrast to PTH 1-34 delivered by liquid nasal spray (90 μg dose) with plasma concentrations only being measured above baseline in two of the seven subjects at 15 and 30 min (14.0–18.9 pg/mL) or 30 and 60 min (17.7–15.4 pg/mL) post-dose administration (for all seven subjects mean C_max_ 5 pg/mL with T_max_ 9 min; t_1/2_ could not be calculated as PTH 1-34 was only measured in the plasma at two time points in these participants, where the concentration was observed to increase with time). The bioavailability of PTH 1-34 after intranasal dosing was approximately 1% (for the subjects with measurable plasma concentrations, but 0.26% if all seven subjects were included in the calculations) compared to the subcutaneous injection of Forsteo^®^. The pharmacokinetic parameters—C_max_, T_max_, AUC_0–2h_ and AUC_0–last_—for the two delivery routes differed markedly (Table 2).

#### 4.1.2. Gamma Scintigraphy

**Figure 2:**
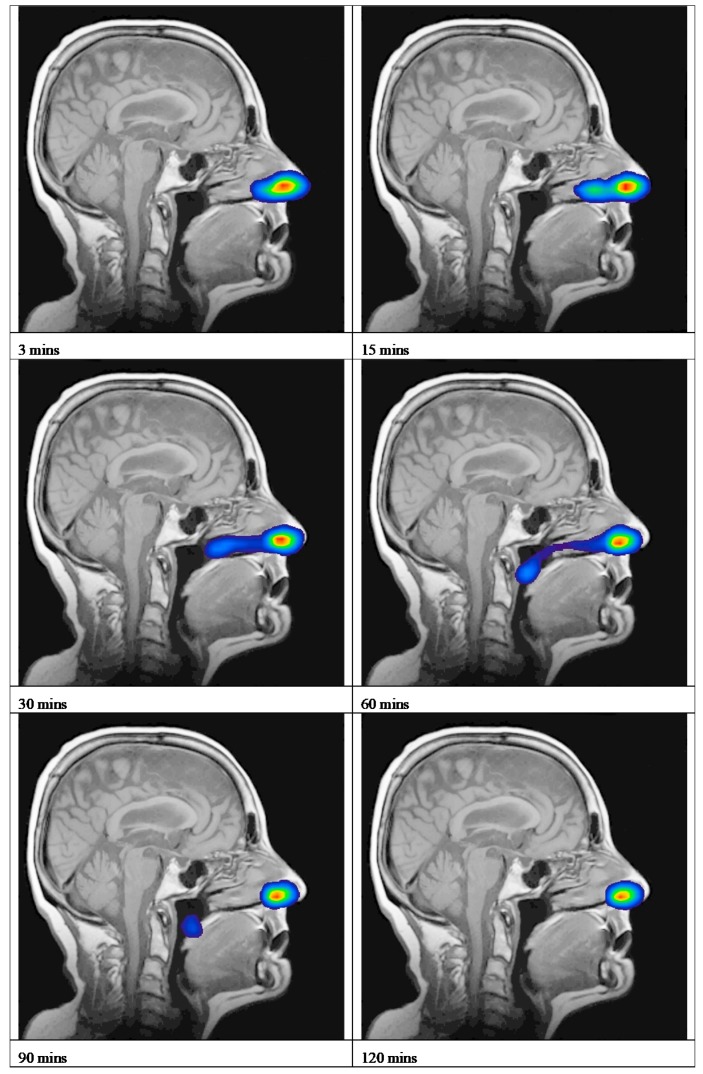
Deposition and clearance of nasal spray formulations delivered using a Rexam SP270 device. Scintigraphic images superimposed upon the MRI sagittal image of Subject 2, showing the clearance of the Tc^99m^-DTPA nasal spray formulation. (The highest levels of activity are shown in red and yellow.).

**Figure 3:**
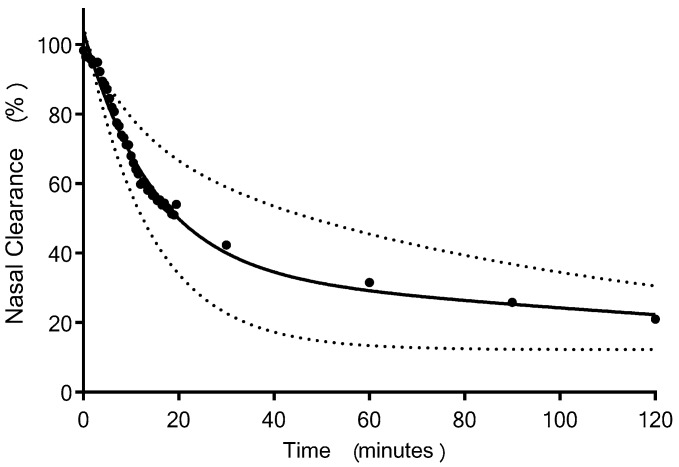
The clearance of Tc-99m-DTPA PTH 1-34 liquid formulation, delivered from a Rexam SP270 nasal spray device fitted with a Rexam 3959 standard winged applicator to healthy female subjects aged over 55 years (two phase decay clearance curve (Prism 7.03) fitted to mean values = solid line, ±95% CI = dotted line, *n* = 7).

An overview of the deposition and clearance of the nasal spray formulation delivered using a Rexam SP270 device is presented as a time series of scintigraphy images (Figure 2 and Supplementary Materials: Section S4). This represents the anatomical location of the Tc-99m-DTPA containing formulation, plotted as a density plot superimposed upon the sagittal view MRI of the participant. These data are a typical example and depicts Subject 2 in this study. The time series of images, 3, 15, 30, 60, 90 and 120 min, illustrates that the formulation was deposited in the anterior of the nasal cavity, extending to the mid-part of the nasal cavity in the region corresponding to the inferior meatus/turbinate, and was subsequently cleared from the inferior turbinates to the nasopharynx. The mean clearance curve obtained for the nasal spray formulation is shown in Figure 3. This depicts the amount of radioactivity (Tc^99m^) that was present in the nasal cavity at each image timepoint. To adjust for variation between subjects in terms of the precise dose of Tc-99m-DTPA deposited, the counts obtained after analysis of the first frame (i.e., after 30 s) were taken as the total counts (100%) for each subject. The scintigraphy clearance data identified that the administered formulation underwent a rapid initial clearance phase (0–20 min) from the nasal cavity. The second phase demonstrated clearance at a slower rate (30–120 min). There was some variability in the clearance data between participants, graphically represented using 95% CI (Figure 3). These data can be summarised as having a median value for T_50%_ of 17.8 min (minimum 10.9, maximum 74.3 min).

#### 4.1.3. Ovine Study

**Figure 4:**
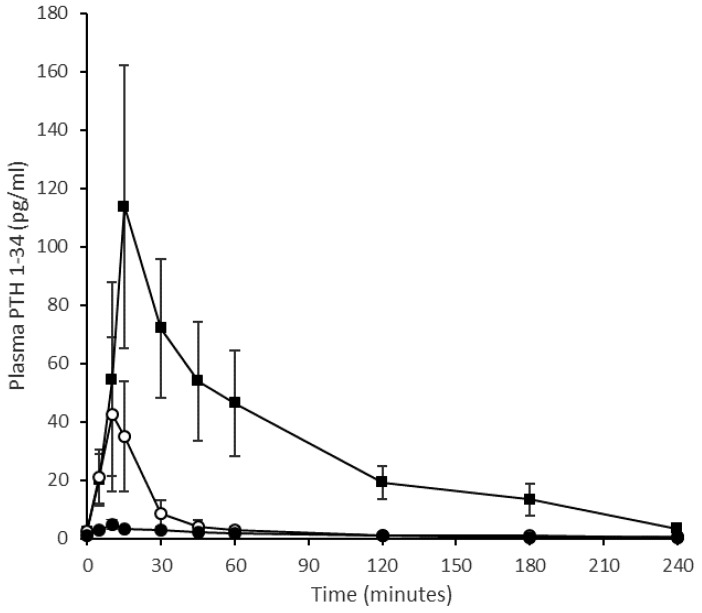
Pharmacokinetic profile of change of plasma PTH (1-34) concentration with time when delivered to an ovine model. Quantification of PTH (1-34) in plasma prepared from blood sampled at 5, 10, 15, 30, 45, 60, 120, 180, 240 min. Squares represent subcutaneous injection (20 μg), open circles represent intranasal liquid formulation (200 μg) and solid circles represent Gellan gum dry powder formulation (130 μg). (Mean values ± SE, *n* = 4 with the exception of the subcutaneous injection where *n* = 3.).

**Figure 5:**
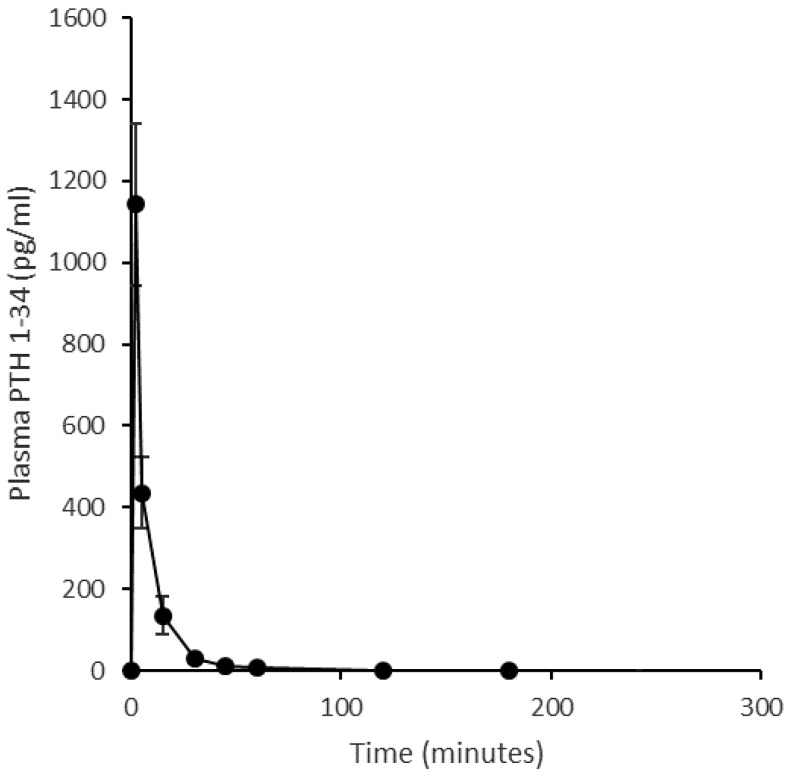
Plasma pharmacokinetic profile of an intravenous injection (20 μg) of PTH (1-34) in an ovine model. Quantification of PTH (1-34) in plasma prepared from blood sampled at 2, 5, 15, 30, 45, 60, 120, 180, 240 min. (Mean ± SE, *n* = 4.).

The mean pharmacokinetic profiles of PTH 1-34 in sheep when delivered nasally, as a liquid nasal spray formulation or dry powder, or by subcutaneous injection is shown in Figure 4. Corresponding summary pharmacokinetic data are presented in Table 3. The ovine study data replicate the clinical study regarding the liquid nasal spray formulation containing Solutol^®^ HS15 making comparison to the subcutaneous injection. As in humans, the subcutaneous profile obtained in sheep showed rapid absorption and elimination of PTH 1-34; C_max_ for the 20 µg subcutaneous injection was 121 ± 47 pg/mL (mean, SE), with a median T_max_ of 10 min (range 5–15 min) and an elimination half-life of 54 ± 8 min (range 43–71 min, *n* = 3). A 200 µg intranasal dose of the PTH 1-34 liquid formulation produced a C_max_ of 47 ± 21 pg/mL and T_max_ of 10 min (range 10–15 min), and concentrations had returned to near baseline values by 30 min post-dose. The dry powder formulation was introduced to the experimental design in an attempt to improve upon the pharmacokinetic profile obtained for the liquid nasal spray. However, this formulation resulted in poor absorption of PTH 1-34 across the nasal mucosa following administration of a nominal 200 µg dose (actual dose based on weighing devices before and after administration ranged from 100–170 µg): C_max_ 8 ± 3 pg/mL. The intravenous injection provided reference pharmacokinetic data and facilitated calculation of absolute bioavailability (Figure 5). The intravenous pharmacokinetic profile of the PTH 1-34 plasma concentration demonstrated rapid elimination of PTH 1-34. The observed C_max_ for a 20 µg dose was 1142 ± 198 pg/mL at 2 min post-injection, returning to near baseline at 30–45 min. The bioavailabilities (F_rel_) of PTH 1-34 from the liquid nasal formulation containing Solutol^®^ HS15 and dry powder formulation, relative to subcutaneous injection, were 1.4 ± 0.3% and 1.0 ± 0.1%, respectively. The absolute bioavailability of subcutaneous injection of PTH 1-34 was 77.0 ± 13.7%.

Monitoring of body weight (Supplementary Materials: Section S3) and adverse effects was conducted during the sheep study. One animal, Sheep 1, was withdrawn from the study prior to dosing on study leg 4 due to the animal suffering what appeared to be a seizure, further details are provided in Supplementary Materials, Section S3. All other general observations with regards to adverse effects monitoring were considered unremarkable.

In terms of monitoring for local tolerability, one incidence of sneezing/snorting was recorded across the four sheep that received the intravenous injection of PTH 1-34, and one incidence across the three sheep that received the subcutaneous injection. After administration of the PTH 1-34 liquid nasal spray formulation, there were six incidences of sneezing/snorting across three of four study animals, compared to ten incidences across three of four study animals after administration of the dry powder formulation. However, 9 of these 16 incidences were in Sheep 1, which, as stated above, was subsequently withdrawn due to ill health. Slight nasal discharge was noted on a single occasion in each of the two sheep that received subcutaneous and intravenous injections. Slight nasal discharge was observed in three of four study animals after intranasal administration of solution (one, two or four incidences observed) and powder (one, two or three incidences) formulations. Similarly, 7 of the 13 incidences of sneezing/snorting in nasally dosed animals were observed in Sheep 1. (Further details relating to incidences of sneezing/snorting and nasal discharge in sheep are summarised in Supplementary Materials, Section S5.)

## 5. Discussion

Currently, in the UK and also globally, bisphosphonates are the first line of drug treatment for osteoporosis in the majority of patients. Subcutaneous injection of teriparatide has received regulatory approval for the treatment of post-menopausal women with osteoporosis, men with hypogonadal or idiopathic osteoporosis, and men and women with glucocorticoid-induced osteoporosis at high risk for fracture. In the UK, this is a second-line treatment in the National Health Service (NHS). At present, the course of treatment is up to 24 months [20]. Teriparatide has an imminent patent expiry date, which is of commercial interest [21]. Greater costs are incurred in the manufacture of injectable formulations compared to those for nasal delivery. An economic consideration regarding teriparatide prescription exists, as it is considerably more expensive than a course of bisphosphonate treatment [22]. Research indicates that patients poorly adhere to the long course of injections prescribed, and therefore a therapeutically efficient simple nasal spray, while cheaper to produce, would also be welcomed by patients being treated with PTH 1-34 [6,7,8].

An absolute bioavailability of 95% (i.e., intravenous injection 100%) has previously been reported for PTH 1-34 in human subjects when administered by subcutaneous injection into the abdominal wall [23]. Our clinical study did not attempt to replicate an assessment of absolute bioavailability, but instead focused on the current clinical route of administration of PTH 1-34 as a comparator for the nasal formulation under investigation, and therefore relative bioavailability to this delivery route. Our study data showed a plasma PTH 1-34 t_1/2_ of 65 min post subcutaneous injection, which is similar to previously published values [19]. C_max_ for this delivery route was 253 pg/mL which is also comparable to those reported [24]. The pharmacokinetics we report for subcutaneous injection represent the consensus option in the literature [25,26,27]. As detailed above, the relative bioavailability of the intranasally dosed liquid nasal spray was approximately 0.26–1% of the subcutaneous injection of Forsteo^®^ (Lilly France S.A.S, France) [28]. Published literature regarding nasal spray formulations are sparse, and those that are available tend to lack formulation and or pharmacokinetic data, making comparison difficult [28,29].

We consider the scintigraphy data to be an important aspect of our study, which demonstrated that the liquid nasal spray was deposited predominantly anteriorly in the nasal cavity. There is sparse literature regarding how the anatomical site of the deposition of the nasal spray formulation within the nasal regions influences transmucosal entry of peptides into the blood [30]. Deposition of the formulation is reported to be required posterior to the nasal valve, possibly within a larger and supra region of the turbinates than we achieved in order to attain optimal absorption of teriparatide [31,32,33,34]. The liquid formulation delivered to the nasal cavity in humans was rapidly cleared, and, hence, the time available for absorption was limited. It is not possible from the scintigraphic images obtained in the study to determine the percentage of formulation that did not reach (and deposit) past the nasal valve. Hence, suboptimal deposition could have contributed to the low bioavailability obtained. Similarly, we do not have data regarding how the excipient polyethylene glycol (15)-hydroxystearate, included in the formulation to promote absorption of PTH 1-34 into the plasma, interacted with the nasal mucosal surface. Perhaps a longer residency could improve drug plasma concentration, and this warrants further research. However, the T_50%_ median clearance time of 17.8 min (minimum 10.9, maximum 74.3 min) is comparable to published data on nasal clearance of liquid formulation [35].

The present clinical study showed disappointing relative bioavailability following delivery in a liquid formulation via the nasal route. This was not consistent with the previous data from our small animal preclinical studies [14]. In the preceding preclinical conscious rat model, we reported the pharmacokinetic performance of an intranasally administered PTH 1-34 subcutaneous formulation, an intranasal liquid formulation with no enhancer and an intranasal solution containing Solutol^®^ HS15. PTH 1-34 doses of 80 μg/kg by subcutaneous injection and 100 μg/kg intranasally were given to rats. The C_max_ and T_max_ were approximately 14 ng/mL at 10 min, with 78% relative bioavailability for intranasally administered PTH 1-34 for the Solutol^®^ HS15-based formulation, compared to 2 ng/mL, 13 min and 8% for the intranasal delivery without enhancer. We therefore expected that a relative bioavailability of at least 10% in humans should have been achievable.

Our sheep pharmacokinetic data for the SC and IV injections of PTH 1-34 are comparable with published values in human subjects. A 20 μg PTH 1-34 subcutaneous dose delivered to post-menopausal women with osteoporosis has been reported in a peer reviewed manuscript by the manufacturers of the commercial formulation to have a C_max_ in the region of 150 pg/mL and an elimination half-life of approximately 1 h. As previously stated, the absolute bioavailability of PTH SC injection in human subjects is approximately 95%. F_ab_ obtained in sheep herein was 77 ± 14% (range 55–108%). The relative bioavailability (F_rel_) of the liquid intranasal formulation in the sheep was 1.4% ± 0.43%, close to that calculated for humans in the clinical study of approximately 1%. The mean C_max_ value in sheep of 47 ± 21 pg/mL can be put in context against reported values for the typical basal endogenous PTH concentration for post-menopausal women (28 pg/mL) and the 65 pg/mL upper limit in premenopausal women for endogenous PTH, yet at the dose given, were below the therapeutic concentrations currently used in treatment of osteoporosis [19,23].

With the aim of improving upon the pharmacokinetics of the liquid nasal spray in humans, our continued research evaluated a bioadhesive powder formulation within a large animal (ovine) model. This is a model known to be highly representative of intranasal drug delivery that closely replicates the clinical findings [17,36]. When using the ovine model to assess the dry powder formulation containing the same absorption enhancer, excipient delivered via the intranasal dry powder formulation did not improve upon the pharmacokinetics of the liquid nasal formulation. Likewise, we have published on the nasal administration in conscious rats of human growth hormone (hGH) (MW 22kDa), a molecular mass approximately five times that of PTH 1-34, with the permeation enhancer Solutol^®^ HS15 (10%) added to the liquid formulation. For the first 2 h after administration, there was a relative bioavailability of 49% [11]. Powder formulations of the hGH with Solutol^®^ HS15 were subsequently administered to human volunteers and compared to a subcutaneous injection of hGH. The relative bioavailability was found to be about 3%, although clinically relevant plasma concentrations could be reached with a twice daily hGF intranasal dose [18].

Observations made during the clinical study with healthy human subjects did not concur with the indicated mild irritation caused to the nasal cavities of the sheep (Supplementary Materials: Section S5) after administration of the nasal formulations [37]. However, the dose of PTH 1-34 in sheep was increased to 200 µg from the 90 µg delivered in the clinical study. A previously published clinical study reported in the literature, where 250, 500 and 1000 µg doses were delivered, reported some symptoms indicative of nasal irritation [28].

## 6. Conclusions

In conclusion, our study shows that PTH 1-34 was poorly absorbed from a liquid nasal spray containing the permeation enhancer Solutol^®^ HS15 in healthy individuals with a 1% relative bioavailability. The scintigraphy imaging confirmed the anatomical site of deposition and rapid clearance of the administered dose in humans. The T_50%_ median was 17.8 min (minimum 10.9, maximum 74.3 min). When increasing the dose in the liquid nasal spray to 200 μg of PTH 1-34, this dose then resulted in a mean C_max_ value of 47 pg/mL in sheep. This value is between one third and one fifth of the C_max_ values for the drug administered at the prescribed dose of 20 μg as a subcutaneous injection for the treatment of osteoporosis in men and women.

The results discussed here for PTH 1-34 in two animal models, rats and sheep, and in humans, together with the results previously reported for hGH in rat and humans, highlights the issue of translation from animal models to humans, not only for the therapeutic effects of drugs but also for the effect of specific drug delivery systems, such as the Solutol^®^ HS15 permeation enhancer. Many nasal delivery systems that include permeation enhancers for peptide drugs have been tested in various animal models, such as rats, rabbits and dogs, where they have been shown to effectively promote the uptake of peptide drugs, only to fail in clinical trials. The translation of effects seen in animal models to humans may depend on many factors, such as the type of intervention, study protocol, animal model and case specific factors. There are opposing opinions in the research community about the relevance of animal models [38]. However from our data, we consider that for peptide nasal delivery, the sheep model provides a better indication of the potential of translation to humans than the rat. Translational research has recently been made a priority both in the US by NIH (budget $500 million per year), in the EU (budget €6 billion) and in the UK (budget £500 million over five years) in order to assess the likelihood of successful cross-species translation [39].

## Figures and Tables

**Table 1:** Dosing of four female sheep (Mule crossbred) with PTH 1-34.

Formulation	Route	Nominal PTH Concentration (μg/mL)	Dose Volume (mL)	Dose Weight (mg)	Nominal PTH Dose (µg)
Solution ^#^	intranasal	1000	0.2 †	not applicable	200
Powder	intranasal	10 **	not applicable	20 † (13 ± 1.5) §	200 (130 ± 15) §
Solution *	subcutaneous	40	0.5	not applicable	20
Solution *	Intravenous	40	0.5 ‡	not applicable	20

^#^ nasal spray formulation as described for clinical study; * injection formulations did not contain 7.5% *w*/*v* polyethylene glycol (15)-hydroxystearate (Ph.Eur.); ** µg/mg; † nasal doses administered via single (right) nostril; ‡ intravenous doses given as a bolus injection; § Mean (± SE) powder and PTH 1-34 dose weights in parentheses calculated by weighing devices before and after dosing.

**Table 2:** Pharmacokinetics of a single dose of an intranasal liquid spray formulation containing the excipient Solutol^®^ HS15 or of a commercially available subcutaneous teriparatide injection formulation.

	Subcutaneous Injection (20 μg Teriparatide) *	Intranasal Spray (90 μg PTH 1-34) ‡
C_max_ (pg/mL)	252.5	5.2
t_max_ (min)	21.4	8.6
t_½_ (min)	64.8	NC
AUC_0–2h_ (pg·min/mL)	17,381.9	242.3
AUC_0–last_ (pg·min/mL)	20,725.6	242.3

*n = 7 volunteers, each dose was given within a cross-over study with a one week washout between doses. ‡ *n* = 7, values were not detectable in 5 of the 7 volunteers. NC: not calculable.

**Table 3:** Summary of ovine pharmacokinetic parameters obtained for PTH 1-34 following intranasal (200 μg dose), subcutaneous (20 μg dose) and intravenous (20 μg dose) administration of PTH 1-34.

	Intranasal Liquid (200 μg)	Intranasal Powder (200 μg ^1^)	Subcutaneous Injection (20 μg)	Intravenous Injection (20 μg)
**C_max_ (pg/mL)**	47 ± 21	8 ± 3	121 ± 47	1142 ± 198 ^2^
**T_max_ (min)**	10 ± 2.0 (10)	11 ± 6.6 (8)	12 ± 1.5 (10)	2 ± 0 (2)
**t_1/2_ (min)**	109 ± 43.7 (77)	96 ± 29.8 (71)	54 ± 8.7 (47)	75 ± 18.1 (78)
**AUC_0-last_ (pg·min/mL)**	1094 ± 410	317 ± 49	7197 ± 2204	10,660 ± 1888
**AUC_INF_ (pg·min/mL)**	1222 ± 370	442 ± 41	7460 ± 2220	10,799 ± 1900 ^3^
**F_rel_ (%) ^4^**	1.4 ± 0.31	1.0 ± 0.12	100	155.8 ± 24.0
**F_ab_ (%) ^5^**	1.1 ± 0.26	0.7 ± 0.03	77.0 ± 13.71	100

Data presented as mean ± SE (median values presented in parentheses where applicable); *n* = 4 except in the subcutaneous injection group, where *n* = 3. Except in the case of F_rel_/F_ab_ values, no correction has been made to the data to account for the dose of PTH 1-34 administered. ^1^ For the powder formulation, this represents the nominal dose; the estimated dose, based on emitted dose weight of powder, was 100 μg Sheep 1, 170 μg Sheep 2, 130 μg Sheep 3 and 120 μg Sheep 4 (mean ± SE 130 ± 15μg); ^2^ C_0_ was 2182 ± 696 pg/mL following extrapolation to *t* = 0; ^3^ Since the subcutaneous injection group only comprised data from three animals (Sheep 2, 3, and 4) the corresponding mean AUC_INF_ ± SE value from these three animals was 9361 ± 1758 pg/mL·min; ^4^ relative to subcutaneous injection for each individual animal, where subcutaneous data were missing (Sheep 1), the average AUC_INF_ (*n* = 3) was used in the calculation (in the case of the intravenous injection, the calculated F_rel_ for *n* = 4 was approximately 156% compared to 140% for *n* = 3, the nominal PTH 1-34 was used throughout except for the powder formulation, where the estimated dose, as given above, was used; ^5^ relative to intravenous injection, the nominal PTH 1-34 dose was used throughout except for the powder formulation, where the estimated dose, as given above, was used.

## Supplementary Materials

The following are available online at https://www.mdpi.com/1999-4923/11/6/265/s1, Section S1: Clinical Trial: Screening Visit Assessment and Familiarisation with Device; Section S2: Clinical Trial: Inclusion and Exclusion Criteria; Section S3: Body Weight of Study Animals and Adverse Event; S4: Formulation Nasal Clearance in Health Volunteers—Clinical Study: Section S5: Nasal Tolerability in Sheep.
