# Supplementary material for: Nasal Administration and Plasma Pharmacokinetics of Parathyroid Hormone Peptide PTH 1-34 for the Treatment of Osteoporosis
Source: Pharmaceutics. 2019 Jun 7;11(6):265. doi: 10.3390/pharmaceutics11060265 (PMC6631119; doi:10.3390/pharmaceutics11060265)
Supplement: Supplementary file 1 [file pharmaceutics-11-00265-s001.zip › pharmaceutics-506903-SI/pharmaceutics-506903-supplementary RGP.docx]

Supplementary Materials: Nasal Administration and Plasma Pharmacokinetics of Parathyroid Hormone Peptide PTH 1-34 for the Treatment of Osteoporosis

Richard G Pearson, Tahir Masud, Elaine Blackshaw, Andrew Naylor, Michael Hinchcliffe, Kirk Jeffery, Faron Jordan, Anjumn Shabir-Ahmed, Gareth King, Andrew L Lewis, Lisbeth Illum and Alan C Perkins

S1. Clinical Trial: Screening Visit Assessment and Familiarisation with Device

• Medical history.

• Demographics (including date of birth, gender, race and ethnicity).

• Physical examination

Cardiovascular System.

Respiratory (including nasal passages).

Abdomen.

Neurological systems.

• Height.

• Weight.

• Body Mass Index.

• Blood pressure (supine).

• Pulse (supine).

• Temperature.

• 12-lead ECG.

• Laboratory safety screens (biochemistry, haematology, and urinalysis).

• Practice dosing with nasal delivery device.

N.B. All laboratory results were be reviewed and the reports signed by with a record filed in the CRF stating whether the results were normal, abnormal but not clinically significant, or abnormal AND clinically significant. In the latter case the eligibility of the participants would have been reviewed.

Tests included HIV, Hepatitis B and C tests and drugs of abuse tests - cannabinoids, cocaine, morphine, benzodiazepines, barbiturates and amphetamines.

S2. Clinical Trial: Inclusion and Exclusion Criteria

Inclusion Criteria

• Be a postmenopausal healthy female and aged greater than 55 years of age.

• Be able to give voluntary informed consent and from whom written consent to participate has been obtained.

• Be able to understand the study, willing to co-operate with the study procedures and able to attend all study assessments.

• Be willing to abstain from alcohol for 24 hours before each dose and until the end of each study day.

• Be willing to abstain from smoking for 24 hours before each dose and until the end of each study day.

• Be willing to avoid caffeine from midnight the evening prior to each study day.

• Concomitant therapies are acceptable and will be reviewed by the Chief Investigator prior to inclusion in the trial

Exclusion Criteria

• Have a history of alcohol or drug abuse and failure of urine tests for drug abuse.

• Have had any investigational drug administered within the previous 3 months.

• Failed to satisfy the investigator’s assessment of fitness to participate based on a completed health screening.

• Have consumed alcohol or tobacco within 24 hours of start of each study day.

• Have consumed caffeinated drink after midnight prior to each study day.

• Have participated in a similar study involving the use of radioisotopes in the previous 3 months such that participating in the current study would exceed the recommended yearly exposure limit (5mSv).

• Have any presently active infectious diseases (such as influenza).

• Have a known hypersensitivity to teriparatide or to any of the excipients in the formulation.

• Have a history of nasal disorders/problems.

• Have a history of allergic rhinitis.

• Have an increased baseline risk of osteosarcoma.

• Have Paget’s disease.

• Have a history of any malignancy or radiotherapy.

• Have a history of diabetes.

• Have a history of hypercalcaemia.

• Are taking any forbidden medications – Digoxin.

• Inability to use both of the intranasal delivery devices.

• Have a condition that prohibits MRI scans

• Have positive HIV or Hepatitis B or C test results or engage in a lifestyle that increases the risk of the possibility of these infections.

S3. Body Weight of Study Animals and Adverse Event

**Table S1. Body Weight and Adverse Event in Sheep**

| **Abbreviated Study Number** | **Body weight (kg)** | | |
| --- | --- | --- | --- |
|  | **10/05/16** | **17/05/16** | **23/05/16** |
| **1** | 42 | 40 | - |
| **2** | 49 | 46 | 47 |
| **3** | 44 | 45 | 45 |
| **4** | 47 | 47 | 47 |
| **Mean ± SD** | 46 ± 3.1  (omitting Sheep 1: 47 ± 2.5 ) | 45 ± 3.1  (omitting Sheep 1: 46 ± 1.0) | 46 ± 1.2 |

Sheep number 1, was withdrawn from the study prior to dosing on Study Leg 4 due to the animal suffering from what appeared to be a seizure. The animal was subsequently euthanized on advice from the NVS/NACWO. The Project Licence Holder (MH) submitted a PPL Standard Condition 18 Report to the Home Office. Collective review of the data concluded that the ‘seizure’ was not dose-related and thus, no remedial action was considered necessary.

S4. Formulation Nasal Clearance in Healthy Volunteers – Clinical Study

S5. Nasal Tolerability in Sheep

**Table S2.** Nasal Tolerability- Incidences of sneezing/snorting and nasal discharge in sheep

| **Treatment Group** | **Sheep**  **Number** | **Potential measures of local tolerance** | |
| --- | --- | --- | --- |
|  |  | **Incidences of sneezing/snorting within the first 60 minutes after dosing** | **Incidences of nasal discharge over the study period** |
| **F1: IN**  **(Nasal Solution)** | 1 | 3 incidences (at 8, 11, 45 min) | At 15-30 min (both nostrils, discharge: slight, clear)  At 45-60 min (right nostril, discharge: slight, clear)  At 120 min (both nostrils, discharge: slight, clear)  At 240 min (right nostril, discharge: slight, clear with pink tinge) |
|  | 2 | 0 | At 0-15 min (right nostril, discharge: slight, clear) |
|  | 3 | 2 incidences (at 9, 17 min) | At 15-30 min (right nostril, discharge: slight, clear)  At 180 min (right nostril, discharge: slight, clear) |
|  | 4 | 1 incidence (at 58 min) | 0 |
|  | **TOTAL** | **6** | - |
| **F2: IN**  **(Nasal Powder)** | 1 | 6 incidences (at 6 (x2), 8, 9, 17, 28 min) | At 0-15 min (right nostril, discharge: slight, clear)  At 15-30 min (right nostril, discharge: slight, clear)  At 30-45 min (right nostril, discharge: slight, clear) |
|  | 2 | 2 incidences (at 39 min) | At 15-30 min (right nostril, discharge: slight, clear)  At 15-30 min (right nostril, discharge: slight, clear) |
|  | 3 | 2 incidences (at 6, 23 min) | At 15-30 min (both nostrils, discharge: slight, clear) |
|  | 4 | 0 | 0 |
|  | **TOTAL** | **10** | - |
| **F3: SC  (Solution for Injection)** | 1 | Not applicable (animal not dosed) | Not applicable (animal not dosed) |
|  | 2 | 0 | At 45-60 min (right nostril, discharge: slight, clear) |
|  | 3 | 1 incidence (at 50 min) | At 0-15 min (both nostrils, discharge: slight, clear) |
|  | 4 | 0 | 0 |
|  | **TOTAL** | **1** | - |
| **F4: IV  (Solution for Injection)** | 1 | 1 incidence (at 16 min) | At 120 min (both nostrils, discharge: slight, clear) |
|  | 2 | 0 | At 180 min (right nostril, discharge: slight, clear) |
|  | 3 | 0 |  |
|  | 4 | 0 |  |
|  | **TOTAL** | **1** | - |
